# Paeoniflorin Protects against ANIT-Induced Cholestatic Liver Injury in Rats via the Activation of SIRT1-FXR Signaling Pathway

**DOI:** 10.1155/2021/8479868

**Published:** 2021-09-02

**Authors:** Lisheng Chen, Shizhang Wei, Honghong Liu, Jianyu Li, Manyi Jing, Yuling Tong, Ruisheng Li, Jianxia Wen, Hanqiu Zhan, Yanling Zhao

**Affiliations:** ^1^Department of Pharmacy, Hebei North University, Zhangjiakou 075000, China; ^2^Department of Pharmacy, The Fifth Medical Center of Chinese PLA General Hospital, Beijing 100039, China; ^3^Integrated TCM & Western Medicine Department, The Fifth Medical Center of Chinese PLA General Hospital, Beijing 100039, China; ^4^Research Center for Clinical and Translational Medicine, The Fifth Medical Center of Chinese PLA General Hospital, Beijing 100039, China; ^5^Department of Pharmacy, Beijing Ditanhospital, Capital Medical University, Beijing 100039, China

## Abstract

Paeoniflorin (PF), a water-soluble monoterpene glycoside, is initially isolated from the dried roots of *Paeonia lactiflora* Pall., which has effects on ameliorating cholestasis in our previous study. However, comprehensive approaches for understanding the protective effects and mechanisms underlying cholestatic liver injury from the regulating of bile acid metabolism have not been sufficiently elucidated. This study was aimed to explore the effectiveness as well as potential mechanism of PF on alpha-naphthylisothiocyanate (ANIT)-induced cholestatic liver injury. Rats with cholestasis induced by ANIT was used to evaluate the protective effects and mechanism of PF by regulating SIRT1/FXR and NF-*κ*B/NLRP3 signaling pathway. Rats were intragastrically administrated with ANIT to establish cholestatic liver injury model. Serum levels of ALT, AST, TBA, TBIL, ALP, *γ*-GT and ALB in rats were detected. The histopathology of the liver of rats was analyzed *in vivo*. The relative mRNA expression and protein expression levels of IL-18, IL-1*β*, TNF-*α*, HO-1, Nrf2, TLR4, NLRP3, Caspase-1, ASC, NF-*κ*B, FXR, and SIRT1 in liver of rats were investigated. The results showed that the serum indexes and the liver histopathology were significantly improved by PF. The overexpression of IL-18, IL-1*β*, TNF-*α*, NLRP3, ASC, and Caspase-1 in liver was markedly reduced by PF. Furthermore, PF dramatically increased the mRNA and protein expressions of SIRT1, FXR, HO-1, and Nrf2, but decreased NF-*κ*B p65 and TLR4 levels in liver of rats. Taken together, the protective effects of PF on cholestatic liver injury were possibly related to the activation of the SIRT1/FXR and inhibition of NF-*κ*B/NLRP3 inflammasome signaling pathway. These findings might provide a potential protection for cholestatic liver injury.

## 1. Introduction

Cholestasis, characterized by bile secretion disorder and excessive bile acid (BA) accumulation in the liver, is clinically associated with a variety of liver diseases, such as progressive familial intrahepatic cholestasis, primary biliary cirrhosis (PBC), primary sclerosing cholangitis (PSC), pregnancy and drug-induced liver injury [1–4]. There are various factors which can lead to cholestasis, such as malnutrition, drug abuse, viral infection and metabolic diseases. The persistent cholestasis can lead to liver fibrosis, cirrhosis, and even liver failure [5–7]. Current opinions believe that oxidative stress, inflammatory damage, and transporter disorders are potential pathological mechanisms related to the development of cholestasis [1, 8]. At present, the clinical treatment of cholestasis is very limited. Both obeticholic acid (OCA) and ursodeoxycholic acid (UDCA) are the therapeutic drugs approved by Food and Drug Administration (FDA), which can be used in the treatment of cholestatic liver diseases. However, the clinical effect is not satisfactory, and approximately 50% of patients show no response to UDCA treatment. In addition, OCA has serious side effects such as abdominal pain, aggravating itching and fatigue [9, 10]. Therefore, it is urgent to find new targets and develop related new candidate drugs to treat cholestatic liver injury.

In recent years, with the vigorous development of complementary and alternative medicine, the enthusiasm for exploring new natural plants for the treatment of cholestatic liver injury has increased exponentially. These agents might provide a complementary therapy and alternative method to enhance the effectiveness of cholestatic liver injury [11]. Paeoniflorin (PF), a water-soluble monoterpene glycoside, is extracted from the dried roots of *Paeonia lactiflora* Pall.. Studies have shown that PF has a wide range of pharmacological effects, such as anti-inflammation [12], anti-oxidation [13], anti-depressant [14], and anti-apoptosis [15]. Our previous studies have shown that PF significantly improve cholestatic liver injury [13, 16]. However, the underlying molecular mechanism in regulating cholestasis and anti-inflammatory has not been fully revealed.

Previous studies have reported the key pathological mechanisms of cholestatic liver injury, providing the possibility for the discovery of new drug candidates for the treatment of cholestasis [17, 18]. Sirtuin 1 (SIRT1) and FXR have been proved to play a central role in protecting cholestatic liver injury [8]. SIRT1, an evolutionarily conserved NAD^+^-dependent histone III deacetylase, is a member of the silent information regulator 2 (Sir2) families of proteins and participates in a wide range of metabolic process including regulating glucose, bile acid, and lipid metabolism as well as reducing oxidative stress and inflammation [19, 20]. SIRT1 directly or indirectly regulates multifarious nuclear receptors and cofactors, such as FXR, LXR, NF-*κ*B, which is considered to be a sensor for various metabolic processes [8, 21, 22]. FXR has been considered as an important nuclear receptor in bile acid metabolism, which plays an important regulatory role in inhibiting BA synthase, restraining liver uptake transporters, inducing bile efflux transporters, and promoting BA metabolism in the liver [17], and currently represents a promising target for new treatments for human cholestatic diseases. Some studies have shown that liver-specific SIRT1 deletion can lead to BA metabolic dysfunction by downregulating the FXR signal, which can be reversed by overexpression of SIRT1 [20].

In this study, based on an assessment of the protective effect of PF on cholestatic liver injury, the effect of PF on SIRT1/FXR and NF-*κ*B/NLRP3 inflammasome signaling pathway was further investigated, which might provide a deeper comprehension of PF for cholestasis.

## 2. Materials and Methods

### 2.1. Materials

Paeoniflorin (PF), with purity higher than 98% determined by ultraperformance liquid chromatography (UPLC) analysis, was purchased from the Chengdu Pufei De Biotech Co., Ltd. (Chengdu, China). ANIT (dissolved in olive oil) was purchased from Sigma Chemical Co. (St. Louis, MO, USA). As the positive control, ursodeoxycholic acid (UDCA) was supplied by Losan Pharma GmbH (Germany). Biochemical indicator kits for alanine aminotransferase (ALT), aspartate aminotransferase (AST), total bilirubin (TBIL), total bile acid (TBA), alkaline phosphatase (ALP), *γ*-glutamyltranspeptidase (*γ*-GT), and albumin (ALB) were obtained from Nanjing Jiancheng Bioengineering Institute (Nanjing, China). All the other experimental supplies were purchased from commercial sources.

### 2.2. Animals and Drug Treatments

Male Sprague-Dawley (SD) rats weighing 200 ± 10 g were purchased from Sibeifu (Beijing) Biotechnology Co., Ltd. (Beijing, China, Permission No. SCXK (jing) 2019-0010). All animals were housed under standard laboratory conditions of temperature (25 ± 2°C) and lighting (12 : 12 h light: dark cycle). Rats were provided with free access to water and chow diet. All animal experiments were approved by the Ethics Committee of the Ethics of Animal Experiments of the Fifth Medical Center of PLA General Hospital (Approval ID: IACUC−2019−004).

All the animals were acclimated for 1 week prior to the experiment. Sixty rats were randomly divided into five groups (12 rats per group), including control group, ANIT group, positive drug group (UDCA, 60 mg/kg), PF low dose group (PFL, 50 mg/kg), and PF high dose group (PFH, 200 mg/kg) [23]. PF and UDCA were dissolved in normal saline and intragastrically given to experimental groups for consecutive five days. At the same time, the control group and ANIT group were intragastrically administrated with the same volume of normal saline. During administration, the control group was administrated with olive oil alone, while the other groups were intragastrically given 60 mg/kg ANIT (dissolved in the olive oil) on the third day to induce cholestatic liver injury. Forty-eight hours after ANIT treatment, all the rats were sacrificed to collect the blood and livers. Blood samples were centrifuged at 3000 ×g for 10 min to obtain serum and stored at −80°C. Liver samples were immediately collected and divided into two parts: one part of liver tissue was excised and fixed in 10% neutral-buffered formalin for HE staining, and another part was snap-frozen in liquid nitrogen and stored at −80°C for RT-PCR, western blotting and immunohistochemistry analysis.

### 2.3. Serum Biochemical Analyses

Synergy H1 Hybrid Reader (Biotech, USA) was used for the detection of serum biochemical indices. The serum levels of ALT, AST, TBIL, TBA, *γ*-GT, ALP as well as ALB were measured using commercial kits in accordance with the manufacturer's instructions.

### 2.4. Histopathological Assessment

After rats were sacrificed, the liver tissues of the same leaf of each rat were immediately collected and fixed in formalin, then embedded in paraffin and sectioned to 5 *μ*m slices. All the slices were stained with hematoxylin and eosin (H&E) following a standard protocol. Histological assessment was carried out independently by two researchers unaware of the different groups. Any differences arising in the process of were settled through discussion and negotiation with another pathologist. Then the pathological changes in the liver tissues were captured with a Nikon microscope (Nikon Instruments Inc., Japan), and the microscope analysis was performed by 200x and 400x.

### 2.5. Quantitative Real-Time PCR

The total RNA rat liver tissue of each group was extracted using Trizol reagent following the manufacturer's instructions. The concentration and purity of the total RNA were determined at 260 nm and 280 nm on a spectrophotometer. Then, cDNA was obtained by reverse transcribed 2 *μ*g of total RNA using a RevertAid First Strand cDNA Synthesis Kit (Thermo Fisher Scientific, MA, USA). The cDNA synthesized was stored at −20°C for subsequent PCR reactions. The amplification reaction of RNA was performed by QuantStudio™ Real-Time PCR System version 1.3 (Applied Biosystems by Thermo Fisher Scientific). The quantity of mRNA was normalized with the GAPDH expression and all the data were calculated for comparison through 2^−∆∆CT^ method. The list of primers used in our study is listed in Table 1.

### 2.6. Western Blotting Analysis

Rat liver tissues (about 80 mg) were homogenized and then lysed in the prepared ice-cold lysis buffer with 1 mM phenylmethylsulfonyl fluoride and a protease inhibitor mixture. Subsequently, tissue debris was removed by centrifugation at 12, 000 ×g and 4°C for 10 min. After centrifugation, the supernatant was aliquoted and stored at −80°C for the subsequent western blotting assay. The concentrations of total protein in supernatants were quantified using a BCA protein assay reagent kit (Beijing Solarbio Science & Technology Co., Ltd, Beijing, China). The samples with the same amount of protein (10 *μ*L) per lane were separated by 10% SDS-PAGE of gel at 80 V for 30 min and 120 V for 1 h. After electrophoresis, the gels were transferred onto polyvinylidene difluoride (PVDF) membranes. All the membranes were blocked with 5% fat-free milk at room temperature for two hours, then incubated overnight at 4°C with antibodies against anti-FXR rabbit polyclonal antibody (bs-12867R, Bioss, dilution: 1 : 1000), anti-SIRT1 rabbit monoclonal antibody (ab189494, Abcam, dilution: 1 : 1000), anit-NLRP3 rabbit monoclonal antibody (ab263899, Abcam, dilution: 1 : 1000), anit-Caspase-1 rabbit polyclonal antibody (342947, ZEN BIO, dilution: 1 : 1000), anit-ASC rabbit polyclonal antibody (340097, ZEN BIO, dilution: 1 : 1000), anit-NF-*κ*B p65 rabbit polyclonal antibody (380172, ZEN BIO, dilution: 1 : 1000), anit-HO-1 rabbit polyclonal antibody (43966, Cell signaling technology, dilution: 1 : 1000), anit-GAPDH rabbit polyclonal antibody (10494-1-AP, proteintech, dilution: 1 : 1000). After washes 5 × 5 min in TBST (Tris-buffered saline with Tween 20), the membranes were incubated with horseradish peroxidase conjugated secondary antibodies (ab6728, abcam, dilution: 1 : 10,000) at room temperature for 1 hour, and subsequently the protein bands were measured using an enhanced chemiluminescence detection system. Samples were assessed for GAPDH content as an internal control.

### 2.7. Immunohistochemical Analysis

The protein levels of NF-*κ*B p65 (380172, ZEN BIO) and Nrf2 (380773, ZEN BIO) were analyzed by immunohistochemistry as previously described [24]. In brief, the liver tissues sections were incubated with primary antibodies directed against NF-*κ*B p65 and Nrf2 overnight at 4°C, then treated with corresponding peroxidase-coupled secondary antibodies for 50 min at room temperature and then developed by diaminobenzidine (DAB). Next, the sections were stained with hematoxylin for 3 minutes. Last, images were captured with a digital camera system under 200x magnification.

### 2.8. Statistical Analysis

All data were presented as the mean ± standard deviation ($\bar{X}\pm \text{SD}$). The differences between the group means were calculated by one-way ANOVA analysis and Duncan's multirange test with the SPSS computer program (version 24.0). GraphPad Prism software (version 8.2.0) was used to visualize the results. The differences were considered to be statistically significant when *P* < 0.05 and highly significant when *P* < 0.01.

## 3. Results

### 3.1. Protective Effect of Paeoniflorin against ANIT-Induced Liver Injury

#### 3.1.1. Effect of PF on Liver Function Indexes

**Figure 1:**
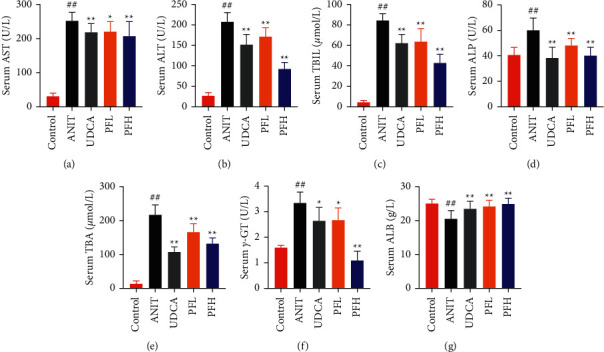
Protective effects of PF on ANIT-induced cholestatic liver injury in rats. Effect of PF on serum levels of AST (a), ALT (b), TBIL (c), ALP (d), TBA (e), *γ*-GT (f), and ALB (g) in ANIT-induced cholestatic liver injury in rats. Data were expressed as mean ± SD. ^#^*P* < 0.05 and ^##^*P* < 0.01 compared with the control group;  ^*∗*^*P* < 0.05 and  ^*∗∗*^*P* < 0.01 compared with the ANIT group (*n* = 6).

As shown in Figures 1(a) and 1(b), serum AST and ALT were increased in the ANIT-treated rats and were significantly reduced by PF pretreatment (*P* < 0.01). Similarly, PF pretreatment also alleviated ANIT-induced cholestatic liver injury, as evidenced by preventing the ANIT-induced the elevation of serum TBIL, ALP, TBA, and *γ*-GT (*P* < 0.01) (Figures 1(c)–1(f)). The content of ALB decreased in ANIT-induced cholestatic rats, compared with the control group. Conversely, PF substantially increased the serum level of ALB (*P* < 0.01) (Figure 1(g)). Taken together, the results indicated that the protective effects of PF on cholestatic liver injury are related to ameliorating liver function, and PF provided remarkable protection against ANIT-induced hepatotoxicity and cholestasis.

#### 3.1.2. Effect of PF on Histopathology

**Figure 2:**
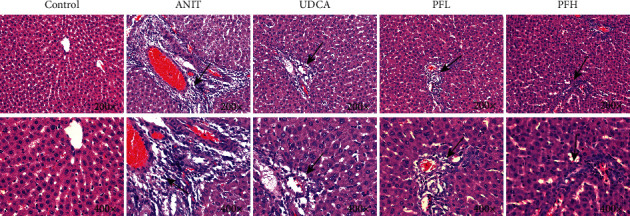
Effect of PF on histological changes in the liver tissue of ANIT-induced cholestatic liver injury in rats (HE stained, 200x and 400x magnification).

H&E staining of liver sections showed that the control group exhibited the normal structure without abnormal morphological changes, liver cell cord in order, sound hepatic cell with uniform stain, and no evidence of infiltration of neutrophilic granulocyte. In contrast, the ANIT-induced group displayed acute infiltration by polymorphonuclear neutrophils, cellular edema, sinusoid congestion, hepatic lobules destruction, and association with hepatic necrosis. However, the liver tissue damage severity significantly relieved in the groups of pretreatment with PF. In the PFL and PFH groups, there was a certain degree of bile duct epithelial damage and defined hepatocyte hydropic degeneration, accompanied by less hepatic neutrophil with infiltration the degree of hepatic necrosis was significantly attenuated and the inflammatory cell infiltration was ameliorated in a dose-dependent manner. Additionally, UDCA, as positive control drug, had an ameliorative effect with liver injury. These results above revealed a significant improvement with cholestatic liver injury in rats pre-treated with PF (Figure 2).

#### 3.1.3. Effect of PF on Inflammatory Factors in the Liver Tissue

**Figure 3:**
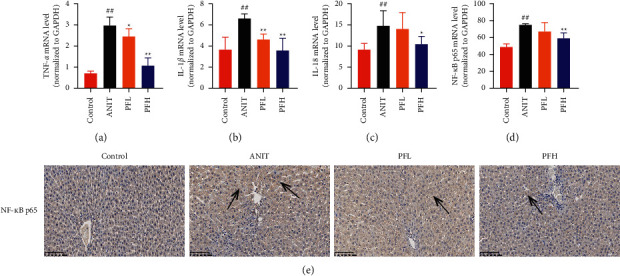
Effect of PF on the mRNA expression of inflammatory factors in rats. (a) Relative mRNA expression of TNF-*α*; (b) relative mRNA expression of IL-1*β*; (c) relative mRNA expression of IL-18. (d) Relative mRNA expression of NF-*κ*B p65; (e) immunohistochemically staining of NF-*κ*B p65. Data were expressed as mean ± SD. ^##^*P* < 0.01 compared with the control group;  ^*∗*^*P* < 0.055 and  ^*∗∗*^*P* < 0.01 compared with the ANIT group (*n* = 6).

**Figure 4:**
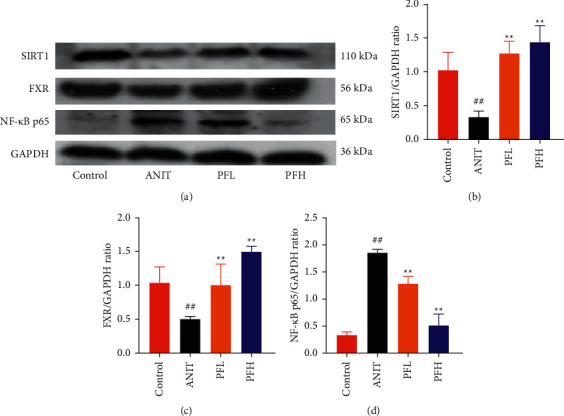
Effect of PF on the protein expressions of SIRT1, FXR, NF-*κ*B p65 in ANIT-induced cholestatic liver injury in rats. (a) Western blot images of SIRT1, FXR, and NF-*κ*B p65; (b) relative protein expression of SIRT1; (c) relative protein expression of FXR; (d) relative protein expression of NF-*κ*B p65. Data were expressed as mean ± SD. ^##^*P* < 0.01 compared with the control group;  ^*∗*^*P* < 0.05 and  ^*∗∗*^*P* < 0.01 compared with the ANIT group (*n* = 3).

Inflammation is one of the characteristics of cholestatic liver injury. Therefore, the mRNA expression level of hepatic inflammation-related factors, including TNF-*α*, IL-1*β*, and IL-18 were determined. As illustrated in Figures 3(a)–3(c) and Table 2, the results indicated that the expression level of TNF-*α*, IL-1*β*, and IL-18 were remarkably increased by ANIT-treated groups (*P* < 0.01). In contrast, PF pretreatment could attenuate the expression of these indicators. To further explore the anti-inflammatory mechanism of PF in cholestasis rats, the mRNA and protein expression level of NF-*κ*B p65, which could modulate various inflammatory factors including TNF-*α*, IL-1*β*, and IL-18 was detected. In this study, the results indicated that the relative mRNA and protein expression of NF-*κ*B p65 were significantly increased in the ANIT-treated group. However, PF significantly inhibited the mRNA and protein expression of NF-*κ*B p65 (Figures 3(d), 4(a), and 4(d)) (*P* < 0.01). In addition, the immunohistochemical analysis was consistent with the result of western blotting analysis (Figure 3(e)). These findings suggest that PF is responsible for inhibiting inflammation in ANIT-induced cholestasis via the NF-*κ*B pathway.

### 3.2. PF Activated SIRT1/FXR Signaling Pathway in Cholestasis Rats

To dissect the potential mechanism of PF for the inhibition of NF-*κ*B pathway, several protein expressions associated with BA homeostasis were investigated. As an important sensor that is critical for bile acid metabolism, the expression of FXR is tightly controlled by an intricate regulatory network in response to various complex environments. Thus, the protein expression of SIRT1, which is the upstream target of FXR, was determined. In comparison to the control group, the protein expression of SIRT1 and FXR decreased markedly in rats treated with ANIT, while their expression in PF-treated rats were restored (Figures 4(a)–4(c)) (*P* < 0.01). These findings suggested that the protective effect of PF against cholestasis might be a result of suppressing NF-*κ*B, which is mediated via SIRT1 and FXR activation.

### 3.3. PF Suppressed the Expression of NF-*κ*B by Activating FXR/Nrf2 Signaling Pathway

**Figure 5:**
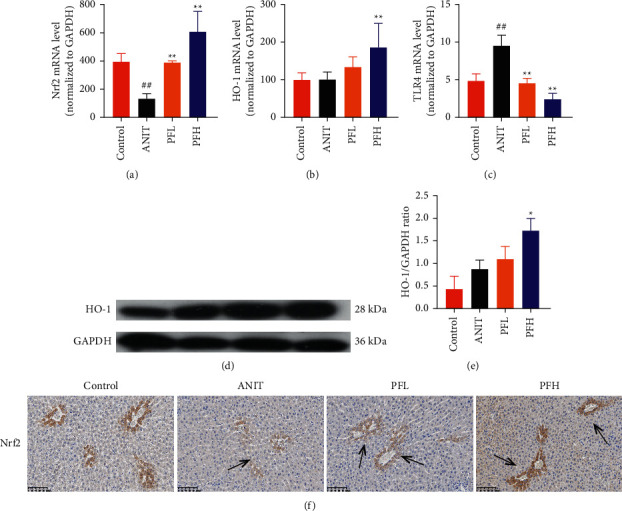
Effect of PF on the expression of FXR/Nrf2 signaling pathway in ANIT-induced rats. (a) The relative mRNA expression of Nrf2; (b) the relative mRNA expression of HO-1; (c) TLR4 mRNA level; (d) western blot images of HO-1; (e) relative protein expression of HO-1; (f) immunohistochemically staining of Nrf2. Data were expressed as mean ± SD. ^##^*P* < 0.01 compared with the control group;  ^*∗*^*P* < 0.05 and  ^*∗∗*^*P* < 0.01 compared with the ANIT group (*n* = 6).

To further explore the mechanism underlying protection of PF against ANIT-induced inflammation, the relative mRNA expressions of Nrf2, HO-1, and TLR4 were determined. The results indicated that ANIT treatment decreased Nrf2 mRNA level (*P* < 0.01), but not significantly altered the relative mRNA expressions of HO-1 (*P* > 0.05). Furthermore, the relative mRNA expressions of TLR4 remarkably increased in ANIT-induced cholestasis rats (*P* < 0.01). As illustrated in Figures 5(a)–5(c), PF pretreatment dramatically upregulated the mRNA expression of HO-1, Nrf2, and downregulated the level of TLR4 (*P* < 0.01). In addition, immunohistochemical staining demonstrated that the increased expression of Nrf2 in ANIT-induced rats after PF pretreatment (Figure 5(f)). Next, the relative protein expression of HO-1 was determined by western blotting analysis. As shown in Figures 5(d) and 5(e), HO-1 was not significantly changed in the ANIT administration group (*P* > 0.05). Conversely, PF therapy increased the relative protein expression of HO-1, especially in high dose of PF (Figures 5(d) and 5(e)) (*P* < 0.01). Overall, the results suggested that PF could exert an anti-inflammatory action on ANIT-induced hepatotoxicity and cholestasis via the FXR/Nrf2 signaling pathway.

### 3.4. PF Inhibited the NF-*κ*B/NLRP3 Inflammasome Pathway in Cholestasis Rats

**Figure 6:**
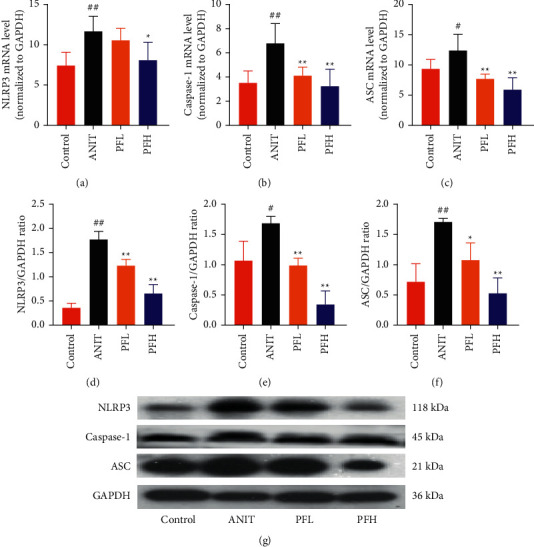
Effect of PF on the relative mRNA and protein expression of the NF-*κ*B/NLRP3 inflammasome pathway in cholestatic liver injury rats induced by ANIT. (a) The relative mRNA expression of NLRP3; (b) the relative mRNA expression of Caspase-1; (c) the relative mRNA expression of ASC (*n* = 6); (d) relative protein expression of NLRP3; (e) relative protein expression of Caspase-1; (f) relative protein expression of ASC; (g) Western blotting images of NLRP3, Caspase-1, and ASC (*n* = 3). Data were expressed as mean ± SD. ^#^*P* < 0.05 and ^##^*P* < 0.01 compared with the control group;  ^*∗*^*P* < 0.05 and  ^*∗∗*^*P* < 0.01 compared with the ANIT group.

To further evaluate the mechanism underlying protection of PF against ANIT-induced cholestatic liver injury, NF-*κ*B/NLRP3 inflammasome pathway was determined. As shown in Figures 6(a)–6(c), the effect of PF on NLRP3 inflammasome mRNA expression, including NLRP3, Caspase-1, and ASC was further detected. As expected, after ANIT stimulation, the relative mRNA expression of NLRP3, Caspase-1, and ASC were increased in the liver tissues compared with that in the control group (*P* < 0.05 or *P* < 0.01). However, PF at the dose of 50 and 200 mg/kg significantly reduced the relative expression of NLRP3, Caspase-1, and ASC in ANIT-induced cholestatic liver injury (*P* < 0.05 or *P* < 0.01). To verify the accuracy of the mRNA results on the induction of these indices by PF, the relative protein levels of NLRP3, Caspase-1, and ASC were measured using western blotting analysis. The results were in consistent with the RT-PCR results (Figures 6(d)–6(g)) (*P* < 0.01). Taken together, these observations showed that PF ameliorated ANIT-induced cholestasis by restraining NF-*κ*B/NLRP3 inflammasome pathway.

## 4. Discussion

**Figure 7:**
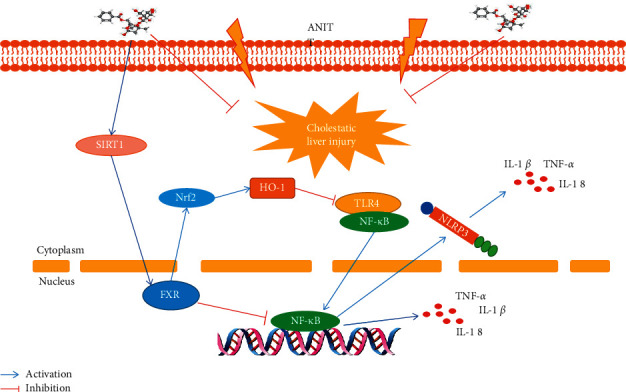
Schematic diagram of molecular biological mechanism of PF in the treatment of cholestatic liver injury.

The present study showed that PF had a good protective effect on cholestatic liver injury induced by ANIT, and the main role of PF on improving cholestatic liver injury were to attenuate inflammation and regulate bile acid metabolism. Further research showed that the protective effect of PF against ANIT-induced cholestatic liver injury by upregulating the expression of SIRT1/FXR and inhibiting NF-*κ*B/NLRP3 inflammasome pathway (Figure 7).

Cholestasis is characterized by intrahepatic accumulation of toxic bile acids due to defective secretion of hepatocellular or cholangio cellular and bile ducts obstruction. Bile acid metabolism disorder and inflammation are known to be the common feature of cholestatic liver injury. The accumulation of bile acids may cause hepatocyte toxicity and liver injury-induced inflammatory response [24–26]. It is believed that inflammation and bile acid metabolism disorder are the decisive generating factors in the pathogenesis of cholestatic liver injury, and anti-inflammatory and regulating bile acid metabolism therapy will be the recommended therapeutic strategy. Up to now, UDCA and OCA are commonly used in the treatment of cholestatic liver diseases, however, the effect are not satisfactory. PF has been proved to have liver protective effect [23]. In our study, ALB, like AST and ALT, is a commonly used clinical indicator of liver function. This study showed that the content of ALB decreased significantly in cholestatic rats, which was in agreement with the previous finding [27]. Pre-administration of PF significantly increased the content of ALB, which provided protection for the liver. In addition, PF significantly decreased ANIT-induced elevation of serum ALT, AST, ALP, TBA, and TBIL levels. Moreover, the pathological injuries were relieved after PF pretreatment at the dose of 50 and 200 mg/kg, which were coincident with the previous report [28].

SIRT1/FXR signaling pathway plays a key role in inflammation and BA metabolism in cholestatic liver injury [29, 30]. One of the interesting findings from the previous study was the significant reduction of SIRT1 in human and mouse cholestasis [31]. In addition, studies have shown that the expression of FXR is suppressed in liver injury, and the activation of FXR has been proved to improve liver injury [8, 32]. Interestingly, it has been found that SIRT1 and FXR can together form an interactive regulatory network [33]. Previous studies have demonstrated that SIRT1 was a critical transcriptional and transactivation regulator of FXR and regulated its activity by deacetylating proteins and histones. More importantly, loss of liver-specific SIRT1 can lead to BA metabolic dysfunction by downregulating FXR signaling, while it can be reversed by SIRT1 overexpression [20, 34]. In addition, activation of the SIRT1/FXR signaling pathway has been confirmed to have a protective effect on cholestatic liver injury [35]. In the current study, our data indicated that SIRT1 and FXR were greatly reduced by ANIT treatment, which were in line with previous studies [25, 36]. While after pretreatment with PF at the dose of 50 and 200 mg/kg, the expression of SIRT1 and FXR were substantially attenuated. Our results suggest that the protective effects of PF against ANIT-induced cholestatic liver injury may be dependent on the activation of the SIRT1/FXR signal.

Interestingly, it has also been shown that activation of FXR exerts anti-inflammatory effects in liver diseases. Accumulating studies have reported that NF-*κ*B is a downstream gene of FXR, and activating FXR signaling pathway had an effective protective effect on liver injury by reducing inflammatory responses [36–39]. In addition to the direct regulation of NF-*κ*B, FXR can also indirectly regulate the expression of NF-*κ*B through Nrf2 [40, 41]. With the discovery of new target genes, it has been found that Nrf2 not only plays a key role in the dynamic balance of redox, but also affects the inflammatory response [42]. Previous studies have shown that Nrf2/HO-1 pathway is considered to be one of the upstream molecules against inflammation induced by NF-*κ*B [42, 43]. Wu et al. recently reported that the lack of Nrf2 effectively prevents PF-mediated inhibition of LPS-induced NF-*κ*B translocation and inflammatory mediator expression [44]. Furthermore, toll-like receptors are important pattern recognition receptors that mediate innate immunity, and their mediating signal pathways play an important role in the occurrence and development of inflammation. The activation of TLR4 can cause the activation of NF-*κ*B into the nucleus. It has previously been reported that the high expression of HO-1 significantly inhibited the expression of TLR4/NF-*κ*B [45]. In the current research, ANIT treatment displayed significant inhibition on Nrf2 expression, but had little effect on the expression of HO-1, along with induction on TLR4 and NF-*κ*B. Moreover, PF pretreatment exhibited an advantageous regulation pattern, which caused the increase of Nrf2 and HO-1, and the decrease of TLR4 and NF-*κ*B. The protective effect of PF in ANIT-induced cholestasis liver injury has been indicated to be due to decreased the levels of NF-*κ*B/TLR4, which is closely related to Nrf2-mediated HO-1 upregulation. Taken together, the above data demonstrate an anti-inflammatory role for PF during cholestatic liver injury, and shed new insights into the significance of the FXR signaling pathway in mediating the protective effect.

The NF-*κ*B/NLRP3 inflammasome pathway is an important pathway for regulating inflammatory factors. Recent studies have shown that NLRP3 may cause liver injury through the activation of NF-*κ*B-related pathways, and NLRP3 inflammasome is of great significance in regulating the occurrence and development of inflammatory response in liver injury [46, 47]. When stimulated by external factors, NF-*κ*B is activated to regulate the expression of NLRP3, IL-18, TNF-*α*, and other genes. Then, macrophages activate the NLRP3-ASC-Caspase-1 inflammasome complex, and the expression of NLRP3 protein regulated by NF-*κ*B provides raw materials for the assembly of the complex. NLRP3 inflammasome activates Caspase-1 via the adaptor protein of ASC, and activated Caspase-1 mediates the maturation of IL-1*β* precursor. Mature IL-1*β* can lead to the aggregation and activation of macrophages in the liver, further amplifying inflammation reaction [48]. With the increase of IL-1*β* and other pro-inflammatory factors, NF-*κ*B is further activated, and the cascade of inflammation is gradually amplified. Eventually, the inflammatory response cannot be controlled and causes severe liver tissue injury. In the current study, the inflammatory factors, including TNF-*α*, IL-1*β*, and IL-18 were significantly elevated in ANIT-treated rats. However, PF reversed the change of inflammatory factors. Furthermore, we found that PF dramatically inhibited the relative protein and mRNA expressions of NLRP3, ASC, and Caspase-1 in ANIT-induced cholestatic liver injury in rats. All the evidence suggest that PF has the ability to alleviate ANIT-induced cholestatic liver injury by negatively regulating inflammation via the NF-*κ*B/NLRP3 signaling pathway.

## 5. Conclusion

In summary, this study demonstrated PF protects against ANIT-induced cholestatic liver injury in rats, and the potential mechanism is related to upregulating the expression of SIRT1/FXR, and inhibiting NF-*κ*B/NLRP3 inflammasome pathway. Thus, PF may be a promising therapeutic agent for the treatment of cholestatic liver disease. These findings may provide evidence why PF has potential protective effects on cholestatic liver injury in the clinic.

## Figures and Tables

**Table 1:** Primers sequences for RT-PCR.

Gene	Forward (5′–3′)	Reverse (5′–3′)
NLRP3	GCAGCGATCAACAGGCGAGAC	TCCCAGCAAACCTATCCACTCCTC
Caspase-1	AAACACCCACTCGTACACGTCTTG	AGGTCAACATCAGCTCCGACTCTC
ASC	TGGTTTGCTGGATGCTCTGTATGG	ACAAGTTCTTGCAGGTCAGGTTCC
NF-*κ*B	GGGATGGCTTCTATGAGGCTGAAC	CTTGCTCCAGGTCTCGCTTCTTC
TLR4	TTGCTGCCAACATCATCCAGGAAG	CAGAGCGGCTACTCAGAAACTGC
HO-1	CAGACAGAGTTTCTTCGCCAGAGG	TGTGAGGACCCATCGCAGGAG
Nrf2	CAAACATTCAAGCCGATTAGAGG	CGGCAACTTTATTCTTCCCTCT
IL-18	CGACCGAACAGCCAACGAATCC	TCACAGATAGGGTCACAGCCAGTC
IL-1*β*	CTCACAGCAGCATCTCGACAAGAG	TCCACGGGCAAGACATAGGTAGC
TNF-*α*	ATGGGCTCCCTCTCATCAGTTCC	GCTCCTCCGCTTGGTGGTTTG
GAPDH	TTCCAGGAGCGAGATCCCGCTAAC	CATGAGCCCTTCCACGATGCCAAAG

**Table 2:** Effect of PF on the relative mRNA expression of TNF-*α*, IL-1*β*, and IL-18 in rats.

Group	TNF-*α*	IL-1*β*	IL-18
Control	0.71 ± 0.11	3.65 ± 1.20	9.11 ± 1.51
ANIT	3.00 ± 0.41^##^	6.60 ± 0.44^##^	14.75 ± 3.60^##^
PFL	2.44 ± 0.37^*∗*^	4.63 ± 0.51^*∗∗*^	14.02 ± 3.88
PFH	1.07 ± 0.36^*∗∗*^	3.58 ± 1.15^*∗∗*^	10.46 ± 1.83^*∗*^

Data were expressed as mean ± SD.^##^*P* < 0.01 compared with control group;  ^*∗*^*P* < 0.05 and  ^*∗∗*^*P* < 0.01 compared with the ANIT group (*n* = 6).

## Data Availability

The data used to support the findings of this study are available from the corresponding author upon reasonable request.

## Abbreviations

PF: Paeoniflorin
ANIT: Alpha-naphthylisothiocyanate
ALT: Alanine aminotransferase
AST: Aspartate aminotransferase
TBIL: Total bilirubin
TBA: Total bile acid
ALP: Alkaline phosphatase
*γ*-GT: *γ*-glutamyltranspeptidase
ALB: Albumin
UDCA: Ursodeoxycholic acid
OCA: Obeticholic acid
BA: Bile acid
PBC: Primary biliary cirrhosis
UPLC: Ultraperformance liquid chromatography
PSC: Primary sclerosing cholangitis
SIRT1: Sirtuin 1
H&E: Hematoxylin and eosin
PVDF: Polyvinylidene difluoride
TBST: Tris-buffered saline with Tween 20.

## References

[1] Wu S.-Y., Cui S.-C., Wang L. (2018). 18*β*-glycyrrhetinic acid protects against alpha-naphthylisothiocyanate-induced cholestasis through activation of the Sirt1/FXR signaling pathway. *Acta Pharmacologica Sinica*.

[2] Zhang X., Ma Z., Liang Q. (2015). Tanshinone IIA exerts protective effects in a LCA-induced cholestatic liver model associated with participation of pregnane X receptor. *Journal of Ethnopharmacology*.

[3] Ashby K., Navarro Almario E. E., Tong W., Borlak J., Mehta R., Chen M. (2018). Review article: therapeutic bile acids and the risks for hepatotoxicity. *Alimentary Pharmacology & Therapeutics*.

[4] Tang X., Yang Q., Yang F. (2016). Target profiling analyses of bile acids in the evaluation of hepatoprotective effect of gentiopicroside on ANIT-induced cholestatic liver injury in mice. *Journal of Ethnopharmacology*.

[5] Chiang J. Y. (2017). Recent advances in understanding bile acid homeostasis. *F1000 Research*.

[6] Geenes V., Chappell L. C., Seed P. T., Steer P. J., Knight M., Williamson C. (2014). Association of severe intrahepatic cholestasis of pregnancy with adverse pregnancy outcomes: a prospective population‐based case‐control study. *Hepatology*.

[7] Meng Q., Chen X.-L., Wang C.-Y. (2015). Alisol *B* 23-acetate protects against ANIT-induced hepatotoxity and cholestasis, due to FXR-mediated regulation of transporters and enzymes involved in bile acid homeostasis. *Toxicology and Applied Pharmacology*.

[8] Gao Z., Zhang J., Wei L. (2020). The protective effects of imperatorin on acetaminophen overdose-induced acute liver injury. *Oxidative Medicine and Cellular Longevity*.

[9] Katsuoka F., Motohashi H., Engel J. D., Yamamoto M. (2005). Nrf2 transcriptionally activates the mafG gene through an antioxidant response element. *Journal of Biological Chemistry*.

[10] Tanaka A., Gershwin M. E. (2017). Finding the cure for primary biliary cholangitisstill waiting. *Liver International*.

[11] Yan J.-Y., Ai G., Zhang X.-J., Xu H.-J., Huang Z.-M. (2015). Investigations of the total flavonoids extracted from flowers of *Abelmoschus manihot* (L.) medic against *α*-naphthylisothiocyanate-induced cholestatic liver injury in rats. *Journal of Ethnopharmacology*.

[12] Hu B., Xu G., Zhang X. (2018). Paeoniflorin attenuates inflammatory pain by inhibiting microglial activation and akt-NF-*κ*b signaling in the central nervous system. *Cellular Physiology and Biochemistry*.

[13] Zhao Y., Zhou G., Wang J. (2013). Paeoniflorin protects against ANIT-induced cholestasis by ameliorating oxidative stress in rats. *Food and Chemical Toxicology*.

[14] Tian D.-D., Wang M., Liu A. (2021). Antidepressant effect of paeoniflorin is through inhibiting pyroptosis CASP-11/GSDMD pathway. *Molecular Neurobiology*.

[15] Zhou D., Zhang S., Hu L. (2019). Inhibition of apoptosis signal-regulating kinase by paeoniflorin attenuates neuroinflammation and ameliorates neuropathic pain. *Journal of Neuroinflammation*.

[16] Chen Z., Ma X., Zhu Y. (2015). Paeoniflorin ameliorates ANIT-induced cholestasis by activating Nrf2 through an PI3K/Akt-dependent pathway in rats. *Phytotherapy Research*.

[17] Gao X., Fu T., Wang C. (2018). Yangonin protects against cholestasis and hepatotoxity via activation of farnesoid X receptor in vivo and in vitro. *Toxicology and Applied Pharmacology*.

[18] Chen J., Raymond K. (2006). Nuclear receptors, bile-acid detoxification, and cholestasis. *The Lancet*.

[19] Sarubbo F., Moranta D., Pani G. (2018). Dietary polyphenols and neurogenesis: molecular interactions and implication for brain ageing and cognition. *Neuroscience & Biobehavioral Reviews*.

[20] Purushotham A., Xu Q., Lu J. (2012). Hepatic deletion of SIRT1 decreases hepatocyte nuclear factor 1*α*/farnesoid X receptor signaling and induces formation of cholesterol gallstones in mice. *Molecular and Cellular Biology*.

[21] Mei Y., Bo L., Hao S. (2020). Isosteviol sodium protects the cardiomyocyte response associated with the SIRT1/PGC-1alpha pathway. *Journal of Cellular and Molecular Medicine*.

[22] Fan W., Zhang R., Han D. (2020). Reduced Sirtuin1 signalling exacerbates diabetic mice hindlimb ischaemia injury and inhibits the protective effect of a liver X receptor agonist. *Journal of Cellular and Molecular Medicine*.

[23] Zhao Y., He X., Ma X. (2017). Paeoniflorin ameliorates cholestasis via regulating hepatic transporters and suppressing inflammation in ANIT-fed rats. *Biomedicine & Pharmacotherapy*.

[24] Yang J., Daochun X., Dong X. (2019). Baicalin protects against 17alpha-ethinylestradiol-induced cholestasis via the sirtuin 1/hepatic nuclear receptor-1alpha/farnesoid X receptor pathway. *Frontiers in Pharmacology*.

[25] Wang T., Zhou Z.-X., Sun L.-X. (2014). Resveratrol effectively attenuates *α*-naphthyl-isothiocyanate-induced acute cholestasis and liver injury through choleretic and anti-inflammatory mechanisms. *Acta Pharmacologica Sinica*.

[26] Xiang D., Yang J., Liu Y. (2019). Calculus bovis sativus improves bile acid homeostasis via farnesoid X receptor-mediated signaling in rats with estrogen-induced cholestasis. *Frontiers in Pharmacology*.

[27] Fahmy S. R., Sayed D. A., Soliman A. M., Almortada N. Y., Aal W. E. A.-E. (2020). Protective effect of Echinochrome against intrahepatic cholestasis induced by alpha-naphthylisothiocyanate in rats. *Brazilian Journal of Biology*.

[28] Zhou H.-Q., Liu W., Wang J. (2017). Paeoniflorin attenuates ANIT-induced cholestasis by inhibiting apoptosis in vivo via mitochondria-dependent pathway. *Biomedicine & Pharmacotherapy*.

[29] Chang H.-C., Guarente L. (2014). SIRT1 and other sirtuins in metabolism. *Trends in Endocrinology and Metabolism*.

[30] Xia Y.-Y., Xu H.-Y., Cai Y.-Y., Si D.-Y., Liu C.-X. (2013). Simultaneous determination of evodiamine and evodine in Beagle dog plasma using liquid chromatography tandem mass spectrometry. *Journal of Asian Natural Products Research*.

[31] Zhao Q., Liu F., Cheng Y. (2019). Celastrol protects from cholestatic liver injury through modulation of SIRT1-FXR signaling. *Molecular & Cellular Proteomics*.

[32] Wei X., Fan X., Feng Z., Ma Y., Lan X., Chen M. (2020). Ethyl acetate extract of herpetospermum pedunculosum alleviates *α*-naphthylisothiocyanate-induced cholestasis by activating the farnesoid x receptor and suppressing oxidative stress and inflammation in rats. *Phytomedicine*.

[33] Kulkarni S. R., Soroka C. J., Hagey L. R., Boyer J. L. (2016). Sirtuin 1 activation alleviates cholestatic liver injury in a cholic acid-fed mouse model of cholestasis. *Hepatology*.

[34] Purushotham A., Schug T. T., Xu Q., Surapureddi S., Guo X., Li X. (2009). Hepatocyte-specific deletion of SIRT1 alters fatty acid metabolism and results in hepatic steatosis and inflammation. *Cell Metabolism*.

[35] García-Rodríguez J. L., Barbier-Torres L., Fernández-Álvarez S. (2014). SIRT1 controls liver regeneration by regulating bile acid metabolism through farnesoid X receptor and mammalian target of rapamycin signaling. *Hepatology*.

[36] Sun S., Zhao B., Qi M. (2020). TUDCA ameliorates liver injury via activation of SIRT1-FXR signaling in a rat hemorrhagic shock model. *Shock*.

[37] Yu J. H., Zheng J. B., Qi J. (2019). Bile acids promote gastric intestinal metaplasia by upregulating CDX2 and MUC2 expression via the FXR/NF-*κ*B signalling pathway. *International Journal of Oncology*.

[38] Zhang Y., Xu Y., Qi Y. (2017). Protective effects of dioscin against doxorubicin-induced nephrotoxicity via adjusting FXR-mediated oxidative stress and inflammation. *Toxicology*.

[39] Bijsmans I. T. G. W., Guercini C., Ramos Pittol J. M. (2015). The glucocorticoid mometasone furoate is a novel FXR ligand that decreases inflammatory but not metabolic gene expression. *Scientific Reports*.

[40] Wu H., Liu G., He Y., Da J., Xie B. (2019). Obeticholic acid protects against diabetic cardiomyopathy by activation of FXR/Nrf2 signaling in db/db mice. *European Journal of Pharmacology*.

[41] Mittal R., Kumar A., Singh D. P., Bishnoi M., Nag T. C. (2018). Ameliorative potential of rutin in combination with nimesulide in STZ model of diabetic neuropathy: targeting Nrf2/HO-1/NF-kB and COX signalling pathway. *Inflammopharmacology*.

[42] Gao Z., Sui J., Fan R., Qu W., Dong X., Sun D. (2020). Emodin protects against acute pancreatitis-associated lung injury by inhibiting NLPR3 inflammasome activation via Nrf2/HO-1 signaling. *Drug Design, Development and Therapy*.

[43] Kim Y. J., Park W. (2016). Anti‐inflammatory effect of quercetin on RAW 264.7 mouse macrophages induced with polyinosinic‐polycytidylic acid. *Molecules*.

[44] Wu X.-X., Huang X.-L., Chen R.-R. (2019). Paeoniflorin prevents intestinal barrier disruption and inhibits lipopolysaccharide (LPS)-Induced inflammation in caco-2 cell monolayers. *Inflammation*.

[45] Baluchnejadmojarad T., Kiasalari Z., Afshin-Majd S., Ghasemi Z., Roghani M. (2017). S-allyl cysteine ameliorates cognitive deficits in streptozotocin-diabetic rats via suppression of oxidative stress, inflammation, and acetylcholinesterase. *European Journal of Pharmacology*.

[46] Xia Y., Wang P., Yan N., Gonzalez F. J., Yan T. (2021). Withaferin A alleviates fulminant hepatitis by targeting macrophage and NLRP3. *Cell Death & Disease*.

[47] Shi Y., Su W., Zhang L. (2020). TGR5 regulates macrophage inflammation in nonalcoholic steatohepatitis by modulating NLRP3 inflammasome activation. *Frontiers in Immunology*.

[48] Broz P., Dixit V. M. (2016). Inflammasomes: mechanism of assembly, regulation and signalling. *Nature Reviews Immunology*.

